# *Bacillus* spp., a bio-control agent enhances the activity of antioxidant defense enzymes in rice against *Pyricularia oryzae*

**DOI:** 10.1371/journal.pone.0187412

**Published:** 2017-11-21

**Authors:** Afroz Rais, Zahra Jabeen, Faluk Shair, Fauzia Yusuf Hafeez, Muhammad Nadeem Hassan

**Affiliations:** Department of Biosciences, COMSATS Institute of Information Technology, Park Road, Islamabad, Pakistan; Universite Paris-Sud, FRANCE

## Abstract

Plant growth promoting rhizobacteria (PGPR) are found to control the plant diseases by adopting various mechanisms. Induced systemic resistance (ISR) is an important defensive strategy manifested by plants against numerous pathogens especially infecting at aerial parts. Rhizobacteria elicit ISR by inducing different pathways in plants through production of various metabolites. In the present study, potential of *Bacillus* spp. KFP-5, KFP-7, KFP-17 was assessed to induce antioxidant enzymes against *Pyricularia oryzae* infection in rice. The antagonistic *Bacillus* spp. significantly induced antioxidant defense enzymes i-e superoxide dismutase (1.7–1.9-fold), peroxidase (3.5–4.1-fold), polyphenol oxidase (3.0–3.8-fold), phenylalanine ammonia-lyase (3.9–4.4-fold), in rice leaves and roots under hydroponic and soil conditions respectively. Furthermore, the antagonistic *Bacillus* spp significantly colonized the rice plants (2.0E+00–9.1E+08) and secreted multiple biocontrol determinants like protease (1.1–5.5 U/mg of soil or U/mL of hydroponic solution), glucanase, (1.0–1.3 U/mg of soil or U/mL of hydroponic solution), siderophores (6.5–42.8 μg/mL or mg) in the rhizosphere of different rice varieties. The results showed that treatment with *Bacillus* spp. enhanced the antioxidant defense activities in infected rice, thus alleviating *P*. *oryzae* induced oxidative damage and suppressing blast disease incidence.

## Introduction

Plant growth promoting rhizobacteria (PGPR) are being widely used as an alternative to chemical fungicides and fertilizers due to their eco- friendly nature [1–4]. They suppress phytopathogens by utilizing various mechanisms in their habitation. Enhanced plant immunity referred as “induced systemic resistance” (ISR) is one of the important mechanism used by various PGPR to secure the plant against extensive range of fungal, bacterial and viral pathogens [5, 6].

ISR is an effective defensive mechanism which is manifested as a result of certain physiological changes in the plant, such as modification in cell wall structure and *de novo* synthesis of antimicrobial compounds like pathogenesis‐related (PR) proteins and phytoalexins, that prevent the dispersion of pathogens [7–11]. The antioxidant enzymes peroxidase (PO), phenylalanine ammonia lyase (PAL) and polyphenol oxidase (PPO) might be elicitors of ISR as their activity in plant has been highly correlated with disease suppression [12].

The activities of various antioxidant enzymes help the plants to mitigate the reactive oxygen species (ROS) level which is source of oxidative stress during pathogen infection [13, 14]. The PO, and PPO enzymes are responsible for the production of phenolic compounds which contribute to the reinforcement of cell barriers [15–18]. PAL initiates the phenylpropanoid pathway, resulting in the biosynthesis of phytoalexins and/or phenolic compounds [19–22]. The phenolic compounds and specific flavonoids induce resistance in host plants after challenge inoculation of pathogens [23–26].

ISR in plants is induced by a number of biotic and abiotic agents. The biotic agents elicit responses around the infected plant cells which cause the cell death through incompatible interactions. Therefore, the pathogen can be trapped in dead cells and seems to be restricted to the initial infection site [27–29].

Several studies show the ability of beneficial microbes to stimulate variety of defense reactions in host plants, in response to pathogen infection, particularly, activity of antioxidant defense enzymes [30–33]. The PGPR bioformulation suppressed the early blight disease in tomato by elevating the activity of antioxidant enzymes such as PAL, PO, PPO, chitinase, β-1,3-glucanase, superoxide dismutase [34], catalase, lipoxygenase, and phenolics [31].

*Bacillus* spp. are recognized as valuable biocontrol agents against diverse phytopathogens through induction of systemic resistance as well as improve efficacy through consistency under field conditions [35, 36]. *Bacillus* spp. produce variable metabolites such as antibiotics, siderophores, salicylic acid (SA), lipopolysaccharides (LPS) and hydrolytic enzymes [37–39] to suppress pathogen either directly or through enhancing the plant defense mechanisms. The involvement of particular strain or/and its metabolites in regulating host defensive ability is scantily understood and needs to be explored. In our previous study, *Bacillus* spp. capable to produce qualitatively higher amounts of siderophores, protease and glucanase *in vitro* significantly reduced the blast disease caused by foliar pathogen *Pyricularia oryzae* [40]. The present study aims to gain an insight into the induction of activity of defense related enzymes in rice and secretion of their potential elicitor in rhizosphere during the *Bacillus* spp. *P*. *oryzae* interaction on different rice varieties.

## Materials and methods

### Microbes and culture conditions

Antagonistic *Bacillus spp*. strains KFP-5 (KT380825), KFP-7 (KT380826) and KFP-17 (KJ719446) capable to control the rice blast caused by virulent strain of *P*. *oryzae* [40] were obtained from Plant Microbe Interaction Laboratory, COMSATS Institute of Information Technology (CIIT) Islamabad, Pakistan. Routinely, the bacterial and fungal strains were grown on Luria-Bertani (LB) broth/agar and potato dextrose (PD) broth/agar respectively.

### Quantification of rhizobacterial bio control determinants

The biocontrol determinates viz siderophores, protease and glucanase produced by *B*. *subtillus* KFP-5, KFP-7 and KFP-17 [40] were quantified by growing them in respective medium as described below:

#### Siderophores

Siderophores produced by the *Bacillus* spp. were quantified [41]. The bacterial strains were grown in the presence and absence of *P*. *oryzae* in nutrient broth at 37°C, 160 rpm for 96 h [42]. The cell free supernatant was collected by centrifugation at 10,000 rpm for 10 min followed by passing through 0.25 μm filter and acidified (pH = 2.0) with 1M HCl. The siderophores were extracted with twice volume of ethyl acetate and dissolved in 5 mL of 50% ethanol. The absorbance was recorded spectrophotometerically at 700 nm. 2, 3- dihydroxy benzoic acid (Alfa Aesar) was used to plot the standard curve. The quantity of siderophore synthesized was expressed as mmol of benzoic acid m/L of culture filtrate. The experiment was repeated thrice to avoid experimental errors.

#### Protease

Protease activity was quantified by the modified method [43]. The bacteria were grown in LB broth [44] and cell free supernatant was obtained as described in section (Siderophores). The supernatant was precipitated by adding 50–55% ammonium sulphate and precipitate was used as crude enzyme extract. The crude enzyme extract and substrate (1.0% casein) solution were mixed in 1:1(V/V) ratio to set up reaction and incubated at 40°C for 20 min. The reaction was stopped by adding 3 mL of 10% tri-chloroacetic acid. The tyrosine released from casein was measured spectrophotometerically (Implen Pearl) at 280 nm and quantified by comparing the standard curve drawn between the absorbance and concentration of tyrosine. The protease activity was calculated by using the following formula [45].

$$\mathrm{Enzymes}\;\mathrm{activity}\;(\mathrm{Units}/\mathrm{mL})=\frac{\mu mole\;tyrosine\;equilent\;release\;\times Total\;Volume\;of\;Assay\;(mL)}{Volume\;of\;enzyme\;taken\;(mL)\times Incubation\;Time\;(min)\times Volumeof\;sample\;taken\;in\;cuvette\;(mL)}$$

#### Glucanase

Glucanase activity of the *Bacillus* spp. was quantified by DNS (Dinitro salicylic acid) method [46]. The DNS method is based on alkaline solution of 3, 5-dinitrosalicylic acid reacts with reducing sugars (eg. Glucose, lactose.) and is converted into 3-amino-5-nitrosalicylic acid with orange color. The bacteria were grown in selective media consisting of different ingredients (g/L) i.e. K_2_HPO_4_ (0.065), KH_2_PO_4_ 117 (0.25), (NH4)_2_SO_4_ (0.05), NaCl (0.25), MgSO_4_.7H_2_O (0.012), yeast extract (0.15), 1% Laminarian at 37°C, 130 rpm for 96 h [47]. The cell free supernatant was obtained as described in section 2.2.1 and used as crude enzyme extract. The reaction was set up by mixing crude enzyme extract and DNS reagent in 1:2 (V/V) followed by incubation in boiling water for 15min. The reaction was stopped by cooling down the mixture. The absorbance was recorded at 500 nm in the concentration of glucose was computed through the standard curve plotted between absorbance and glucose concentration. The glucanase activity was measured by using the formula mentioned in section (Protease) except μmole glucose was used instead of tyrosine [46].

### *In planta* experiments

Ability of *Bacillus* spp. to induce activity of defense related enzymes and produce biocontrol determinants *in situ* was assessed on three rice varieties viz Basmati-515, Basmati-super and Basmati-385 under hydroponic and soil conditions. The seeds were surface sterilized with 0.1% mercuric chloride (HgCl_2_) and soaked in sterile distilled water for 12–16 h. The imbibed seeds were placed in rows on sterilized sand in a tray for germination and growth until 12–15 days.

#### Experimental design and microbial inoculation

The experiment was designed in complete randomized design (CRD) with three replications and six treatments viz *P*. *oryzae* (T_1_ = Negative control), = Fungicide + *P*. *oryzae* (T_2_ = Chemical control), *Bacillus* sp KFP-5 + *P*. *oryzae* (T_3_), *Bacillus* sp KFP-7 + *P*. *oryzae* (T_4_), *Bacillus* sp KFP-17 + *P*. *oryzae* (T_5_), Untreated (T_6_). The bacterial and fungal inoculum was applied as described in our previous study [40].

#### Growth of rice plants in hydroponic culture

Twelve days old seedling roots were sterilized with mercuric chloride (0.1%), dipped in bacterial suspension (10^9^ CFU/mL) for 2 h and transplanted in hydroponic tray filled with the nutrient solutions consisting of (NH_4_)_2_ (48.2 mg/L), MgSO_4_ (154.88 mg/L), K_2_SO_4_ (15.9 mg/L), KNO_3_ (18.5 mg/L), KH_2_PO_4_ (24.8 mg/L), Ca (NO_3_)_2_ (86.17 mg/L), Fe- citrate (7 mg/L), MnCl_2_.4H_2_O (0.9 mg/L), ZnSO_4_.7H_2_O (0.11 mg/L), CuSO_4_ 5H_2_O (0.04 mg/L) and H_2_MoO_4_ (0.01 mg/L)[48]. Fungicide was applied by dipping the seedling roots in solution consisting of 1.5g Thiophanate Methyl dissolved per liter of water. Hydroponic solution was replaced after every four days. The cells present in nutrient solution were harvested by centrifugation and re dissolved in the fresh nutrient solution every time.

#### Growth of rice plants in soil

The earthen pots (20x 30 cm^2^) were obtained from local Nursury and filled with sterilized soil. The soil has clay loam texture with total nitrogen (N = 0.04%), phosphorous (P = 15.7 mg/Kg), sodium (Na = 4.6 meq/L),calcium magnesium (CaMg = 7.4meq/L), zinc (Zn = 0.76), iron (Fe = 4.73 mg/Kg), electric conductivity (1.3 dms^-1^), pH (7.81), organic matter (0.72%), sodium adsorption ratio (SAR 2.6), exchangeable sodium percentage (ESP 2.5).The rice plants were grown by following the recommended agronomic practices as describe in our pervious study. [40].

### Quantification of antioxidant defense related enzymes activity

The antioxidant defense related enzymes viz superoxide dismutase [34], peroxidase (PO) [49], polyphenol oxidase (PPO) and phenylalanine ammonialyas (PAL) were quantified from rice plants grown in hydroponic and pot conditions after 96 h of fungal inoculation.

The representative samples of leaves and roots were collected from rice plants. Two gram of each sample was crushed with 4 mL of 0.1M phosphate buffer (pH 7) in pre chilled mortar and pestle. The homogenate was centrifuged at 10,000 rpm, 4°C for 15 min and supernatant was used as crude enzyme extract [50]. Absorbance of PO, PPO and PAL, was observed on spectrophotometer at 280 nm, 450nm, 460 nm respectively [8, 38, 51–54]. Enzyme activity was expressed as change in absorbance min/ g fresh weight (FW).

For SOD activity the supernatants were obtained in Phosphate buffers (pH 7). Total SOD activity was assayed by the photochemical [55]. One unit of enzyme activity was defined as the amount of enzyme required for 50% inhibition of the rate of NBT reduction measured at 560 nm (U/ g FW) [56].

### Colonization of *Bacillus* spp. with rice rhizosphere/rhizoplane/endosphere

The colonizing ability of *Bacillus* spp with root rhizosphere /rhizoplane/endosphere of rice was assessed at 96^th^ h of pathogen inoculation. The rhizosphere/rhizoplane/endosphere sample of rice plants growing in hydroponic solution was obtained by collecting the different roots i.e. primary, secondary and lateral and 20 mL nutrient solution associated with these roots using sterile syringe [57, 58]. The roots were grinded, homogenized in nutrient solution and 1mL of this homogenate was used for further process. In pot experiment, the roots and adhering soil were collected and pooled. One g of homogenized soil was used to isolate the rhizobacteria. The *Bacillus* spp. were isolated by serial dilution as described in our previous studies [37, 38, 40].

### Quantification of biocontrol determinants secreted *in situ*

Siderophores, protease and glucanase enzymes secreted by rice plants were extracted from the hydroponic solutions and soil. Hydroponic solution (50 mL) was centrifuged and supernatant was passed through 0.25μm syringe filter. Cell free solution was used for quantification of biocontrol determinants.

For extraction of siderophores, glucanase and protease from soil, One g soil was suspended in 100 mL of phosphate buffer (1 M, pH = 7.0), mixed well by shaking at 200 rpm for 10 min and kept at room temperature until settling down of the soil particles [59]. The mixture was filtered through Whatman filter paper three times. The filtrate was centrifuged at 13,000 rpm for 5 min at 4°C [60]. The biocontrol determinants were extracted and quantified from these supernatants as described in section (Siderophores)

### Statistical analysis

The data was analyzed by variance analysis [61] using the statistical package Statistix 8.2. All the minimal data set has been included in supplementary material (S1 File).

## Results

### Quantity of biocontrol determinants produced by *Bacillus* spp

**Fig 1:**
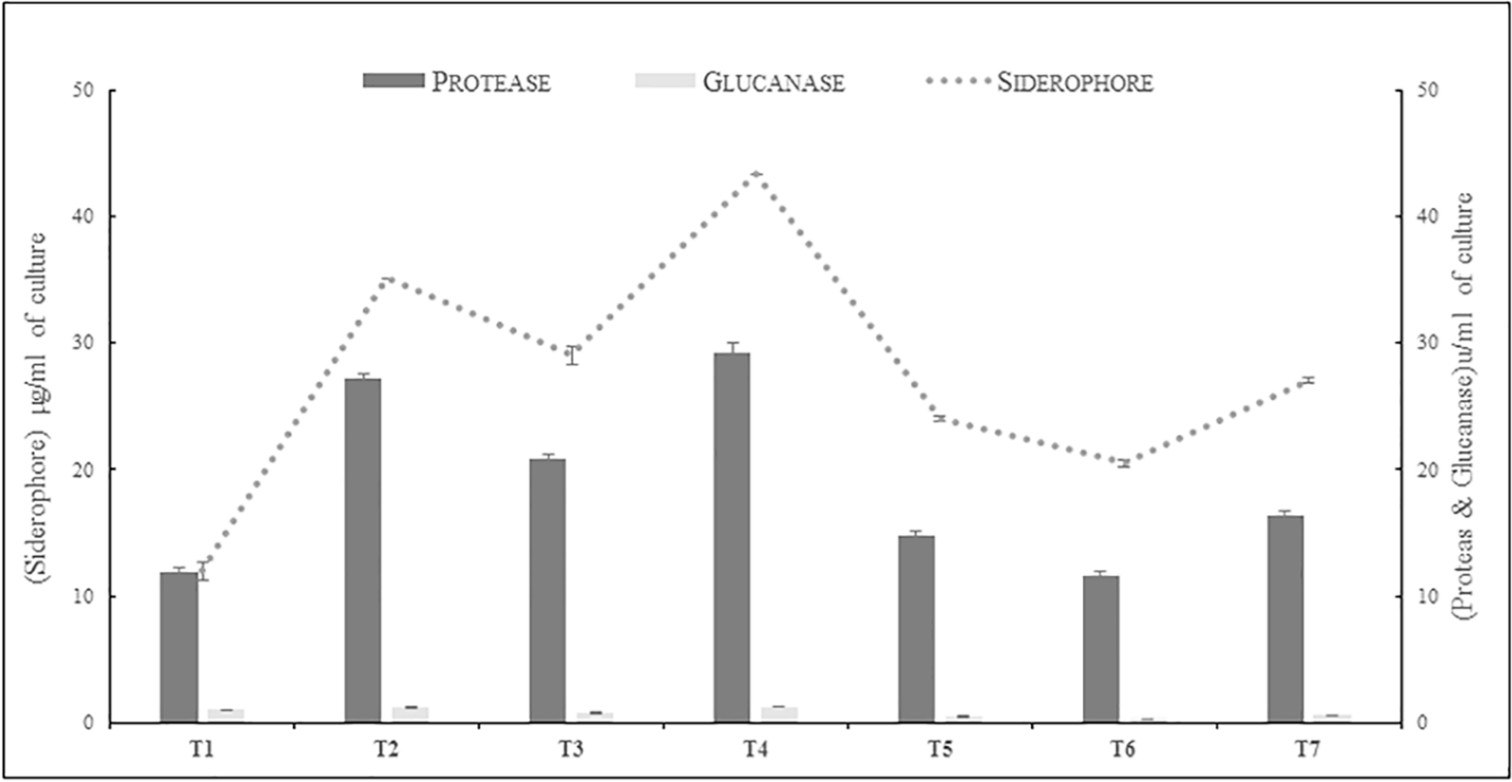
Production of biocontrol determinants by antagonistic bacteria in presence and absence of *P*.*oryzae*. T1 = *P*.*oryzae*, T2 = *Bacillus* sp KFP-5+*P*.*oryzae*, T3 = *Bacillus* sp KFP-7+*P*.*oryzae*, T4 = *Bacillus* sp KFP- 17+*P*.*oryzae*, T5 = *Bacillus* sp KFP-5, T6 = *Bacillus* sp KFP-7.T7 = KFP- *Bacillus* sp 17. Values are mean of three replicates Bars represent the standard error of means. All treatments are significantly different from each other at P<0.001.

*Bacillus* spp. strains KFP-5, KFP-7 and KFP-17 produced variable quantities of siderophore, protease and glucanase in absence as well as presence of rice blast pathogen *P*.*oryzae*. The maximum biocontrol determinants were produced in presence of *P*.*oryzae* i-e., siderophore (29–43.3μg/mL), protease (20.9–29.3U/mL) and glucanase (0.73–1.24 U/mL). However in absence of *P*.*oryzae*, the production of biocontrol determinants was lower i-e., siderophores (20.5–27.0 μg/mL), protease (11.6–24 U/mL) and glucanase (0.3–0.6 U/mL). The *Bacillus* spp. showed same order of biocontrol determinants production in the absence or presence of *P*. *oryzae* i-e KFP-17>KFP-5>KFP-7 (Fig 1).

### Effect of *Bacillus* spp. on the activity of antioxidant defense enzymes in rice

The antioxidant defense enzyme SOD, POD, PPO, PAL which act as first line of defense against the plant pathogens were elicited by the antagonistic *Bacillus* spp. The tested strains enhanced enzymatic activity in leaves and roots of three tested rice varieties viz—basmati super, basmati-515, basmati-385 grown in hydroponic solutions as well as soil.

#### Superoxide dismutase (SOD) activity

**Fig 2:**
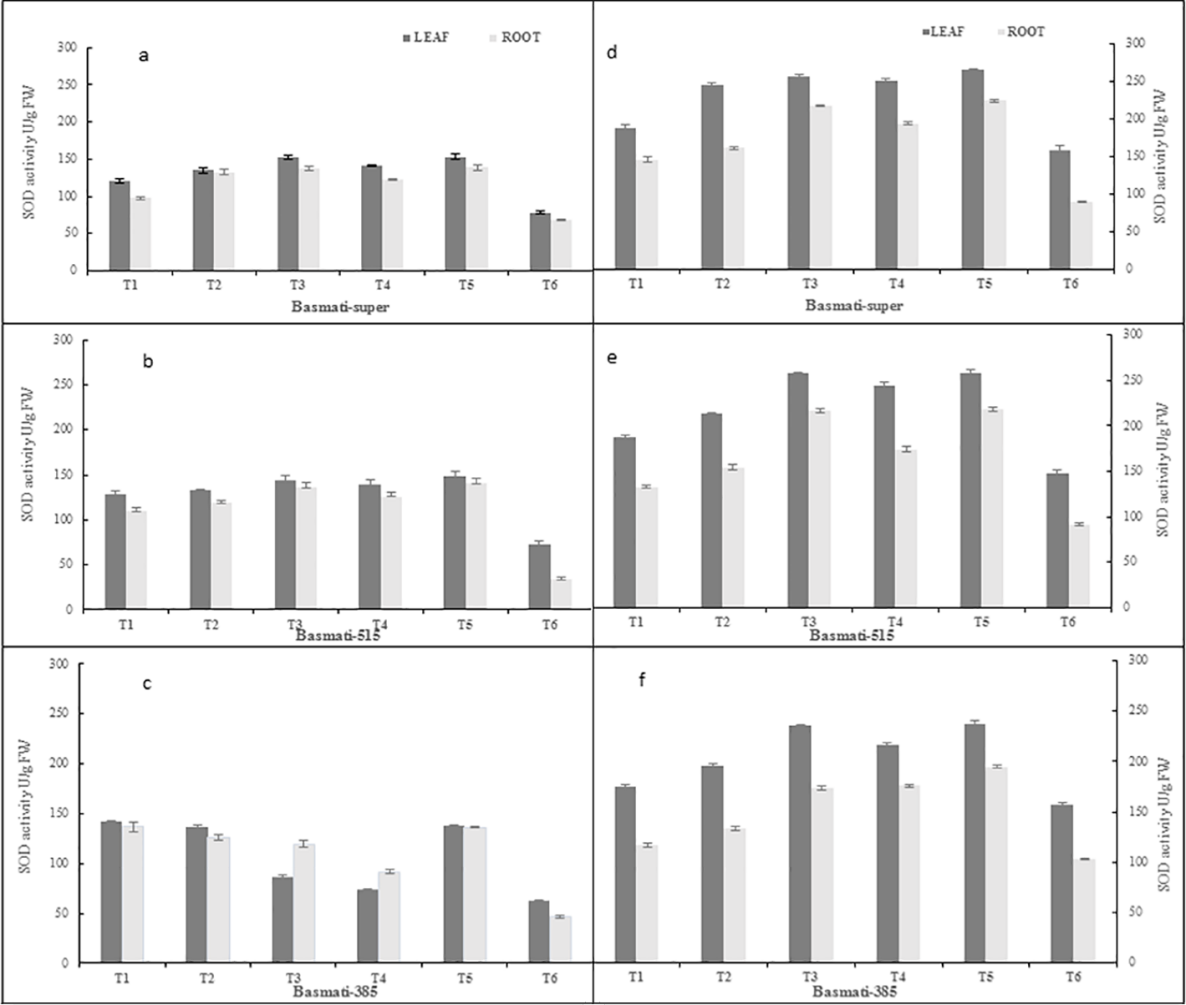
SOD contents in leaves and roots of different rice varieties grown hydroponicaly (left panel a, b, c and pot/soil experiment (right panel d, e,f). T1 = *P*.*oryzae*, T2 = Fungicide +*P*.*oryzae*, T3 = *Bacillus* sp KFP-5+ *P*.*oryzae*, T4- *Bacillus* sp KFP-7+ *P*.*oryzae* T5 = *Bacillus* sp KFP-17+ *P*.*oryzae* T6 = untreated. Values are means of three replicates and vertical bars represent the standard error. All treatments are significantly different from each other at P<0.001.

The antagonistic bacteria induced the SOD activity (1.7–1.9 fold) over negative control (*P*. *oryzae*) in both hydroponic and soil conditions. The induction of SOD activity in leaves was higher (1.3–1.9 fold) as compare to that of roots (1.1–1.7 fold) in all varieties.Maximum SOD activity was observed in basmati-385 (1.8–1.9 fold) followed by that of basmati-515 (1.2–1.6 fold) and basmati-super (1.2–1.4 fold). *Bacillus* sp KFP-17 elicited highest SOD activity (1.2–1.9 fold) followed by that of *Bacillus* sp KFP5 and *Bacillus* sp KFP-7 (1.1–1.8 fold). The SOD activity was little induced in fungicide treatment (1.0–1.2 fold). The SOD activity was highly inducted in pot experiments (1.9 fold) as compare to that of hydroponic (1.4 fold) experiment (Fig 2).

#### Peroxidase (PO) activity

**Fig 3:**
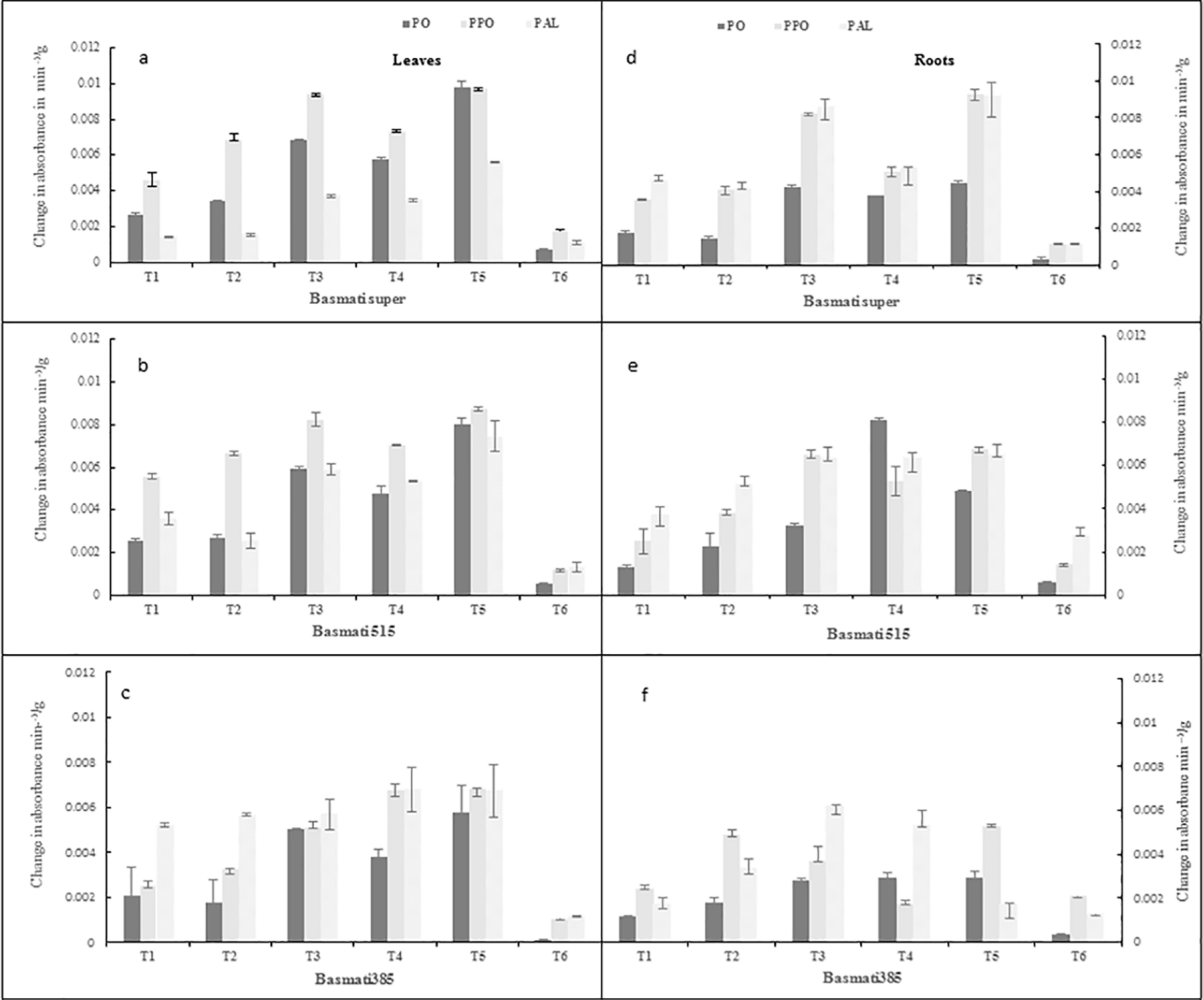
**Defense enzyme (PO, PPO, PAL) contents in leaves (left panel a, b,c) and roots (right panel d,e,f) of rice varieties grown hydroponicaly.** T1 = *P*.*oryzae*, T2 = Fungicide +*P*.*oryzae*, T3 = *Bacillus* sp KFP-5+ *P*.*oryzae*, T4- *Bacillus* sp KFP-7+ *P*.*oryzae* T5 = *Bacillus* sp KFP-17+ *P*.*oryzae* T6 = untreated. Values are means of three replicates and vertical bars represent the standard error. All treatments are significantly different from each other at P<0.001.

**Fig 4:**
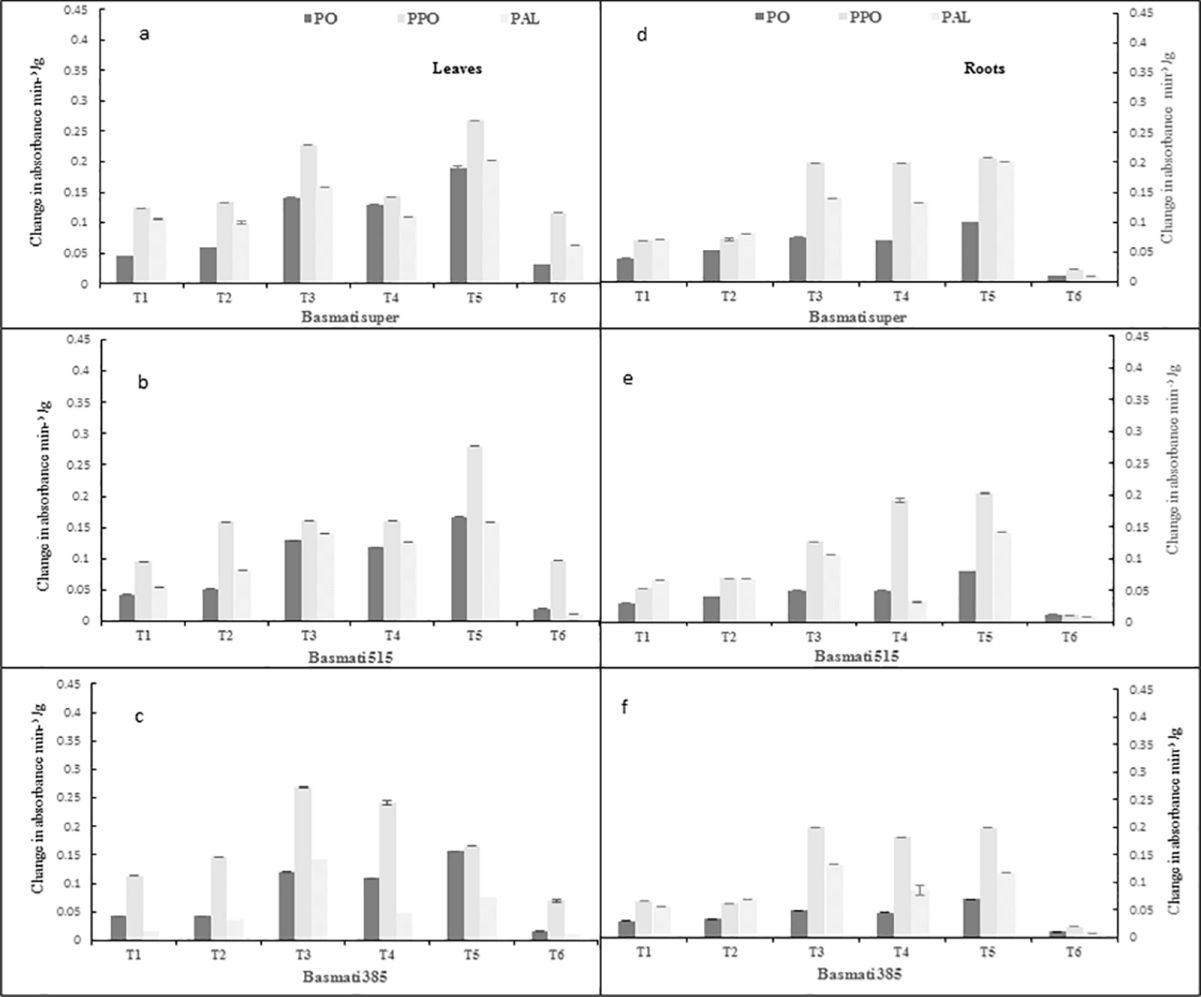
**Defense enzyme (PO, PPO, PAL) contents in leaves (left panel a, b,c) and roots (right panel d,e,f) of rice varieties grown in pot/soil.** T1 = *P*.*oryzae*, T2 = Fungicide +*P*.*oryzae*, T3 = *Bacillus* sp KFP-5+ *P*.*oryzae*, T4- *Bacillus* sp KFP-7+ *P*.*oryzae* T5 = *Bacillus* sp KFP-17+ *P*.*oryzae* T6 = untreated. Values are means of three replicates and vertical bars represent the standard error. All treatments are significantly different from each other at P<0.001.

In present investigation, the antagonistic bacteria induced the POD activity up to (3.5–4.1 fold) over negative control (*P*. *oryzae*) in both conditions. The expression level of POD activity was comparatively higher in leaves (3.5–4.2 fold) than roots (2.0–4.1 fold). The variety basmati-super showed the maximum response (3.5–4.1 fold) followed by that of basmati -515 (3.2–3.9 fold) and basmati -385 (2.8–3.6 fold). The maximum induction was showed in *Bacillus* sp KFP-17 treated plants (3.5–4.1 fold) followed by that of *Bacillus* sp KFP-5 (2.4–3.2 fold) and *Bacillus* sp KFP-7 (2.1–2.9 fold). POD activity of fungicide was observed (1.1–1.3 fold) over negative control. The POD activity was highly inducted in pot experiments (4.2 fold) as compare to hydroponic experiment (3.6 fold) as shown in (Figs 3 and 4).

#### Polyphenol oxidase (PPO) activity

The antagonistic bacteria induced the maximum PPO activity (3.0–3.8 fold) over negative control (*P*. *oryzae*) in hydroponic and soil conditions. The bacteria enhanced the activity of PPO in roots (1.2–3.8 fold) as compared to leaves (1.5–2.9 fold) in all varieties. The PPO activity was highest in basmati -super (1.2–3.8 fold) followed by that of basmati-385 (1.5–3.0 fold) and basmati-515 (2.8–2.9 fold) while maximum in *Bacillus* sp KFP-17 (2.2–3.8 fold) followed by that of *Bacillus* sp KFP-5 (1.9–3.0 fold) and *Bacillus* sp KFP-7(1.2–1.7 fold). The PPO activity was observed in fungicide (1.1–1.3 fold) over negative control. The PPO activity was highly inducted in pot experiments (3.8 fold) as compare to hydroponic experiment (2.9 fold) as shown in (Figs 3 and 4).

#### Phenyalanin ammonia-lyase (PAL) activity

In present analysis, the PAL activity was induced by the antagonistic bacteria (3.9–4.4 fold) in both conditions. The expression level of PAL activity was considerably higher in leaves (3.9–4.4 fold) as compared to roots (1.6–2.4 fold). Among rice varieties, highest response was observed in basmati-385 (3.9–4.4 folds) followed by that of basmati-515 (2.2–2.9 fold) and basmati-super (1.0–1.9fold). *Bacillus* sp KFP-17 induced highest PAL activity (1.9–4.4fold) followed by that of *Bacillus* sp KFP-5 (1.5–3.8 fold) and *Bacillus* sp KFP-7 (1.0–2.9 fold). The fungicide also induced PAL activity (1.0–2.1 fold) over negative control. The PAL activity was highly inducted in pot experiments (4.4-fold) as compare to hydroponic experiment (1.9-fold; Figs 3 and 4).

### *In situ* secretion of biocontrol determinants

Offensive PGPR colonization and defensive retention of rhizosphere niches are enabled by production of bacterial biocontrol determinants. The antagonistic bacteria secreted detectable quantities of siderophores, protease and glucanase in the rhizosphere of rice plants grown hydroponically and soil.

#### Protease

**Fig 5:**
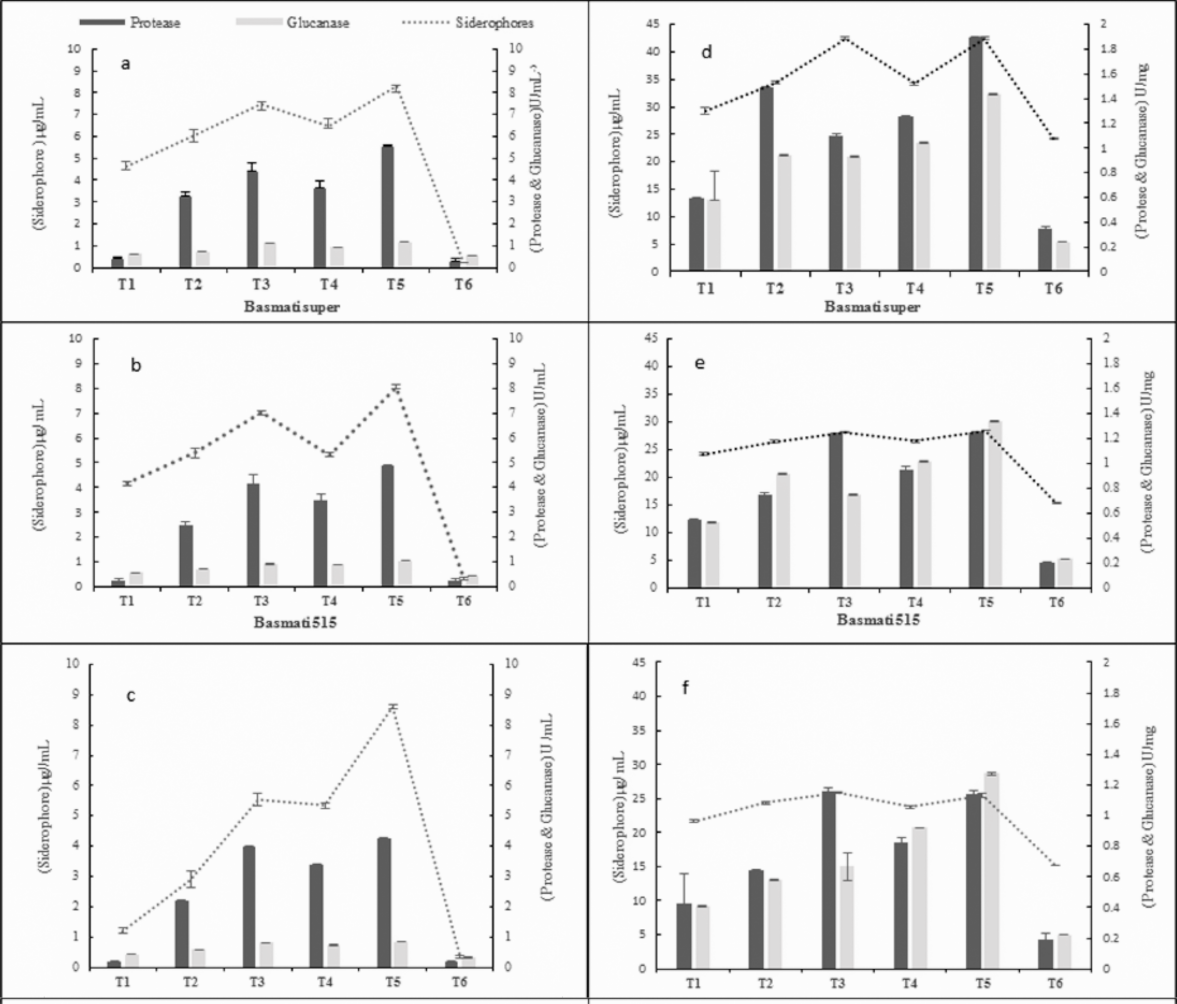
**Secretion of biocontrol determinants in rhizosphere of different rice varieties grown hydroponically (left panel a, b, c) and pot/ soil (right panel d, e, f).** T1 = *P*.*oryzae*, T2 = Fungicide +*P*.*oryzae*, T3 = *Bacillus* sp KFP-5+ *P*.*oryzae*, T4- *Bacillus* sp KFP-7+ *P*.*oryzae* T5 = *Bacillus* sp KFP-17+ *P*.*oryzae* T6 = untreated. Values are means of three replicates and vertical bars represent the standard error. All treatments are significantly different from each other at P<0.001.

The antagonistic strains secreted protease in rhizosphere of all rice varieties. The maximum protease activity was recorded in rhizosphere of basmati -super (1.1–5.5 U/mg of soil or U/mL of hydroponic solution) followed by that of basmati-515 (1.0–4.9 U/mg or U/mL) and basmati-385 (0.8–4.2 U/mg or U/mL).The *Bacillus* sp KFP-17 secreted the maximum protease (1.1–5.5 U/mg or U/mL) followed by that of *Bacillus* sp KFP-5 (1.1–4.4 U/mg or U/mL) and *Bacillus* sp KFP-7(0.8–3.6 U/mg or U/mL). The minor quantity of protease was also recorded from the rhizosphere of fungicide treated plants (0.2–0.6 U/mg or U/mL). Higher amount of protease was recorded in the rhizosphere of soil grown rice plants (5.5 U/mg) as compare to that of grown hydroponically (1.9 U/mL) as shown in (Fig 5).

#### Glucanase

The glucanase was also secreted in the rhizosphere of all rice varieties. The maximum glucanase production was recorded in basmati-super (1.0–1.3 U/mg of soil or U/mL of hydroponic soluotion) followed by that of basmati-515 (0.8–1.3 U/mg or U/mL) and basmati -385 (0.7–1.3 U/mg or U/mL).The *Bacillus* sp KFP-17 secreted maximum glucanase (0.8–1.3 U/mg or U/mL) followed by that of *Bacillus* KFP-5 sp (0.7–1.1 U/mg or U/mL) and *Bacillus* sp KFP-7 (0.7–1.0 U/mg or U/mL). The production of glucanase (0.3–0.6 U/mg or U/mL) was observed in fungicide treated plants. Higher amount of glucanase (1.3 U/mg) was recovered from the rhizosphere of rice plants grown in soil as compare to that of grown in hydroponic (1.2 U/mL) (Fig 5).

#### Siderophores

The antagonistic strains also secreted siderophores in the rice rhizosphere. Among rice varieties, maximum siderophore activity was observed in basmati-super (6.5–42.8 μg/mL) followed by that of basmati-515 (6.7–28.4 μg/mL) and basmati-385 (3.6–25.8 μg/mL).The *Bacillus* sp KFP-17 secreted highest amount of siderophore (5.53–42.5 μg/mL) followed by that of *Bacillus* sp KFP-5 (4.4–42.0 μg/mL) and *Bacillus* sp KFP-7(3.6–34.2 μg/mL). The siderophores (2.9–29.2 μg/mL) were recovered in fungicide treatment. Higher quantity of protease (42.5 μg/mL) was secreted in soil as compared to hydroponic (6.7 μg/mL) (Fig 5).

### Root colonization by antagonistic bacteria

The antagonistic strains significantly colonized the rice plants grown either hydroponically or soil. The maximum root colonization (2.0E+00–9.1E+08) was observed in *Bacillus* sp KFP-17 colonized (6.3E+07–9.13E+08) followed by *Bacillus* sp KFP-5 (4.8E+07–8.4E+08) and *Bacillus* sp KFP-7(4.3E+07–6.13E+08). Furthermore the rice varieties basmati-super (8.6E+08–9.1E+08) followed by that of basmati -515 (7.4E+07–8.4E+08) and basmati -385 (6.6E+06–7.8E+08). The root colonization is shown in (Tables 1 and 2).

**Table 1:** Potential of *Bacillus spp* to colonize root under hydroponic conditions.

Treatment	Bas-super	Bas-515	Bas-385
*P*.*oryzae*	3.3E+01^c^	2.6E+03^d^	2.0E+00^c^
Fungicide +*P*.*oryzae*	3.5E+01^bc^	2.67E+02^d^	7.67E+01^b^
*Bacillus* sp KFP-5*+P*.*oryzae*	8.2 E+08^b^	6.3E+07^b^	4.8E+07^b^
*Bacillus* sp KFP-7*+P*.*oryzae*	6.1E+08^b^	4.3E+07^c^	4.5E+07^c^
*Bacillus* sp KFP-17*+P*.*oryzae*	8.6E+08^a^	7.4E+07^a^	6.3E+07^a^
Untreated	6.67E-01^c^	6.67E-01^d^	6.67E-01^c^

Values are means of three replicates and values bearing different letters in the same column are significantly different from each other according to the Fisher’s least significant difference (LSD) test at p<0.001.

**Table 2:** Potential of *Bacillus spp* to colonize root under pot/soil conditions.

Treatment	Bas-super	Bas-515	Bas-385
*P*.*oryzae*	4.67E+01^c^	3.33E+02^c^	7.00E+01^b^
Fungicide *+P*.*oryzae*	6.00E+02^c^	4.00E+02^c^	7.00E+01^b^
*Bacillus* sp KFP-5*+P*.*oryzae*	8.4E+08^ab^	7.8E+08^a^	5.2E+08^ab^
*Bacillus* sp KFP-7*+P*.*oryzae*	5.0E+08^b^	4.7E+08^b^	4.7E+08^ab^
*Bacillus* sp KFP-17*+P*.*oryzae*	9.13E+08^a^	8.47E+08^a^	7.8E+08^a^
Untreated	1.67E+00^c^	1.33E+00^c^	1.33E+00^b^

Values are means of three replicates and values bearing different letters in the same column are significantly different from each other according to the Fisher’s least significant difference (LSD) test at p <0.001.

## Discussion

Use of antioxidant mechanism by plants in protection from aerial phytopathogens has been well documented [62–65]. Several PGPR stimulate the antioxidant machinery in plants but whether they secrete the eliciting determinants *in situ* has not yet been reported. In this study, we unrevealed the activation of antioxidant system in rice by the antagonistic *Bacillus* sp in response to blast pathogen *P*. *oryzae* through the quantification of defense enzymes involved in induction of systemic resistance in the host. These findings clearly demonstrate that *Bacillus* sp enhanced the activity of peroxidase [49], polyphenol oxidase (PPO), peroxidase, phenylalanine ammonia lyase (PAL) and superoxide dismutase [34] by secreting protease, siderophores and glucanase near rice rhizosphere grown in hydroponic as well as soil.

*P*. *oryzae* infection in rice generates reactive oxygen species (ROS), such as radicals of superoxide (O_2_^•-^), hydroxyl (^•^OH) and molecules of hydrogen per oxide (H_2_O_2_) [66–68]. The accumulation of ROS cause damage in the infected plant cells [69, 70]. The effect of ROS molecules are neutralized by an effective ROS-scavenging system which majorly comprise of antioxidant enzymes such as superoxide dismutase, peroxidase, catalase, Ascorbate peroxidase (APX) Guaicol peroxidase (GPX) Monodehydroascorbate reductase (MDHAR) Dehydroascorbate reductase (DHAR) Glutathione reductase (GR) and polyphenol oxidase (PPO), phenylalanine ammonia lyase (PAL) [34].

Our results depict that plants inoculated with *P*. *oryzae* in absence of biocontrol agents showed a decreased activity of PO, SOD, PPO and PAL, confirming that oxidative damage is associated with the ROS scavenging system. Low amounts of antioxidant enzymes are produced under high level of ROS [71, 72].

Nevertheless, the antagonistic *Bacillus* sp significantly enhanced the activity of antioxidant enzymes by approximately 2–5 fold in shoots and roots of rice in response to *P*. *oryzae* infection. These findings are in consistent with the earlier reports in which potential of antagonistic bacteria to enhance activities of antioxidant enzymes in rice as defensive mechanism to multiple pathogens has been reported [12, 73–75]. However, in our study, strain KFP-17 was best in eliciting the activity of PO, PPO and SOD. This enhanced activity may be correlated with its best ability to suppress the blast disease as reported in our previous studies [40]. Moreover, a positive correlation between production of antifungal metabolites; siderophores, protease and glucanase by the *Bacillus* sp. and activity of antioxidant enzymes was observed (Tables 3 and 4). These findings are further supported by the earlier reports in which siderophores and hydrolytic enzymes production by rhizobacteria significantly enhance the activity of antioxidant enzymes. A significant decrease in disease incidence/progression of *Sclerotium rolfsii* pathogen was observed attributable to high activity of PO, PAL and PPO in rice plants treated with siderophore producing rhizobacteria/*Streptomyces* spp.[76]. Similar findings have been documented by Naureen et al [77] in which hydrolytic enzymes producing rhizobacteria induced systemic resistance against rice sheath blight disease in rice caused by the causative agent *Rhizoctonia solani*. Similarly, there is increasing evidence depicting that siderophores, protease and cellulase producing microbes protect the plants from pathogen stress by eliciting the ROS scavenging system [63, 72, 78].

**Table 3:** Pearson^,^ s correlation among antioxidant enzyme content and biocontrol determinants secrected in rice rhizosphere grown hydroponicaly.

Basmati Super							
	Glucanase	PAL	PO	PPO	Protease	Siderophores	SOD
**Glucanase**	1.00**						
**PAL**	0.940**	1.00					
**PO**	0.988**	0.910**	1.00				
**PPO**	0.984**	0.955**	0.956**	1.00			
**Protease**	0.972**	0.937**	0.966**	0.944**	1.00		
**Siderophores**	0.959**	0.969**	0.949**	0.947**	0.939**	1.00	
**SOD**	0.996**	0.921**	0.987**	0.977**	0.956**	0.946**	1.00
**Basmati 515**							
**Glucanase**	1.00						
**PAL**	0.927**	1.00					
**PO**	0.893**	0.990**	1.00				
**PPO**	0.940**	0.965**	0.919**	1.00			
**Protease**	0.989**	0.943**	0.902**	0.972**	1.00		
**Siderophores**	0.878**	0.802**	0.801**	0.763*	0.825**	1.00	
**SOD**	0.917**	0.971**	0.937**	0.981**	0.957**	0.720*	1.00
**Basmti 385**							
**Glucanase**	1.00						
**PAL**	0.914**	1.00					
**PO**	0.227	0.178	1.00				
**PPO**	0.36	0.507*	0.066	1.00			
**Protease**	0.995**	0.906**	0.252	0.329	1.00		
**Siderophores**	0.984**	0.899**	0.324	0.415	0.979**	1.00	
**SOD**	0.991**	0.894**	0.231	0.296	0.996**	0.964**	1.00

Asterisks indicate significance as follows p< 0.05

**Highly significant

* Significant

**Table 4:** Pearson^,^ s correlation among antioxidant enzyme content and biocontrol determinants secrected in rice rhizosphere grown in pot/soil.

Basmati Super							
	Glucanase	PAL	PO	PPO	Protease	SOD	Siderophores
**Glucanase**	1.00						
**PAL**	0.933**	1.00					
**PO**	0.969**	0.936**	1.00				
**PPO**	0.923**	0.985**	0.965**	1.00			
**Protease**	0.868**	0.916**	0.957**	0.970**	1.00		
**SOD**	0.886**	0.839**	0.968**	0.910**	0.962**	1.00	
**Siderophores**	0.827**	0.943**	0.909**	0.977**	0.972**	0.883**	1.00
**Basmati 515**							
**Glucanase**	1.00						
**PAL**	0.687	1.00					
**PO**	0.797**	0.829**	1.00				
**PPO**	0.718**	0.903**	0.979**	1.00			
**Protease**	0.968**	0.837**	0.832**	0.801**	1.00		
**SOD**	0.657	0.977**	0.897**	0.964**	0.794**	1.00	
**Siderophores**	0.658	0.978**	0.847**	0.922**	0.798**	0.982**	1.00
**Basmati 385**							
**Glucanase**	1.00						
**PAL**	0.660*	1.00					
**PO**	0.739*	0.948**	1.00				
**PPO**	0.735*	0.934**	0.999**	1.00			
**Protease**	0.579	0.986**	0.922**	0.907**	1.00		
**SOD**	0.740*	0.985**	0.986**	0.978**	0.959**	1.00	
**Siderophores**	0.556	0.977**	0.904**	0.889**	0.958**	0.948**	1.00

Asterisks indicate significance as follows p≤ 0.05

**Highly significant

* Significant

Our findings firstly present the secretion of siderophores, protease and glucanase in the rhizosphere of rice. A significant correlation between the quantities of protease, siderophores, glucanase recovered from rhizosphere and activities of antioxidant enzymes indicate the role of these metabolites in activation of antioxidant enzyme system under *P*. *oryzae* infection. The role of these metabolites in eliciting systemic resistance against *Fusarium oxysporum*, *Pyricularia oryzae*, *Alternaria* sp. and *Sclerotium* sp. has been established earlier [79, 80]. It has been reported that strain *P*. *fluorescens* 3551, B224 was unable to activate defense enzymes /induce SR/ suppress pathogen, when it was mutated to enable its siderophore producing ability. Similarly, mutated *B*. *subtilis* strain deficient in chitinase production showed less antagonistic and hydrolytic activity [80, 81].

Our findings indicate their role in disease management not only by suppressing the pathogen but also through modulating the ROS scavenging system in plants. This mechanism of defense is highly worth full for the control of diseases caused by the aerial pathogens where the chances of direct contact between the pathogen and biocontrol agent are very rare. Furthermore, studies indicate that activities of enzymes involved in the biosynthesis of either flavonoids or structural polyphenols such as lignin have also been induced by the application of PGPR [82–85]. Here, we observed that application of antagonistic *Bacillus* sp. (especially the strain KFP-17) enhanced the PPO and PAL activity in rice plants. PAL is a first enzyme of phenylpropnoid pathway and involved in the biosynthesis of liginin, phenolics, phytoalexins and salicylic acid which serve as first defense line against phytopathogens [86–89].

Differential changes in the activity of POD and SOD, as a consequence of *Bacillus* spp. treatments, were also observed. SOD constitutes the first line of defense in the enzymatic antioxidant responses by catalyzing the dismutation of O_2_^•-^ to H_2_O_2_ and O_2_ [90, 91]. The H_2_O_2_ is further scavenged by the POD. Major differences were detected in the antioxidant enzyme of roots/shoots and treatments (bacteria) under *P*. *oryzae* stress (Fig 5D and 5F).

There are numerous studies indicating the ability of PGPR to induce antioxidant activity in plants but the exact mechanism by which they induce is still a question. It could be speculated that PGPR, being microbes, contain conserved signatures on their cell wall or motility organs. Plant recognizes them through its efficient immune system consisting of pattern recognition receptors (PRRs) and microbe associated molecular patterns (MAMPs). This PRR-mediated microbe sensing induces various defense responses including activation of antioxidant enzymes [92].

Microbes secrete different secondary metabolites such as effectors, proteases or other metabolites to colonize the host [93]. The PGPR metabolites especially hydrolytic enzymes cleave their substrates yielding oligosaccharides/ sugars which act as signal to induce antioxidant enzymes [94]. The siderophores also trigger signaling mechanism by altering the iron status in plant vascular system leading to the higher induction of antioxidant enzymes [95].

The different trends in soil and hydroponic treatments suggest that there are fundamental differences in the nature of these two systems that influence the responses of defense enzymes. The production of antioxidant enzyme and biochemical determinants was lower in hydroponic conditions as compared to pot experiments. Selected rice variety basmati-super showed maximum activity of antioxidant enzymes and biochemical determinants with respect to all treatments as compared to the basmati -515 and basmati- 385. Accordingly, further work should focus on the exploration of physiological mechanisms in this variety responsible for higher response to antagonistic bacteria inoculation.

## Supporting information

S1 File (XLSX)

## References

[1] Hussain S, Siddique T, Saleem M, Arshad M, Khalid A. Impact of Pesticides on Soil Microbial Diversity, Enzymes, and Biochemical Reactions. Advances in Agronomy 2009;102:159–200.

[2] Thakur A. Evaluation of biological control strategies against a range of plant pathogens. 2017.

[3] Antoun H, Prévost D. Ecology of plant growth promoting rhizobacteria PGPR: Biocontrol and biofertilization: Springer; 2005 p. 1–38.

[4] Reddy M, Ilao RI, Faylon PS. Recent Advances in Biofertilizers and Biofungicides (PGPR) for Sustainable Agriculture: Cambridge Scholars Publishing; 2014.

[5] Vallad GE, Goodman RM. Systemic acquired resistance and induced systemic resistance in conventional agriculture. Crop Sci 2004;44:1920–34.

[6] Shafi J, Tian H, Ji M. Bacillus species as versatile weapons for plant pathogens: a review. Biotechnology & Biotechnological Equipment 2017;31:446–59.

[7] Kuc J. Phytoalexins, stress metabolism, and disease resistance in plants. Annu Rev Phytopathol 1995;33:275–97. doi: 10.1146/annurev.py.33.090195.001423 18999962 10.1146/annurev.py.33.090195.001423

[8] Hammerschmidt R. Phytoalexins: what have we learned after 60 years? Annu Rev Phytopathol 1999;37:285–306. doi: 10.1146/annurev.phyto.37.1.285 11701825 10.1146/annurev.phyto.37.1.285

[9] Conrath U, Thulke O, Katz V, Schwindling S, Kohler A. Priming as a mechanism in induced systemic resistance of plants. Eur J Plant Pathol 2001;107:113–9.

[10] Walters D, Newton A, Lyon G. Induced resistance: helping plants to help themselves. Biologist 2005;52:28‐33.

[11] Filippi MCC, Da Silva GB, Silva-Lobo VL, Côrtes MVC, Moraes AJG, Prabhu AS. Leaf blast (*Magnaporthe oryzae*) suppression and growth promotion by rhizobacteria on aerobic rice in Brazil. Biol Control 2011;58:160–6.

[12] Yasmin S, Zaka A, Imran A, Zahid MA, Yousaf S, Rasul G, et al Plant Growth Promotion and Suppression of Bacterial Leaf Blight in Rice by Inoculated Bacteria. PloS one 2016;11:e0160688 doi: 10.1371/journal.pone.0160688 27532545 10.1371/journal.pone.0160688 PMC4988697

[13] Shi Q-H, Zhu Z-J, Juan L, Qian Q-Q. Combined effects of excess Mn and low pH on oxidative stress and antioxidant enzymes in cucumber roots. Agricultural Sciences in China 2006;5:767–72.

[14] Liu TT, Wu P, Wang LH, Zhou Q. Response of soybean seed germination to cadmium and acid rain. Biol Trace Elem Res 2011;144:1186–96. doi: 10.1007/s12011-011-9053-6 21479540 10.1007/s12011-011-9053-6

[15] Souguir D, Ferjani E, Ledoigt G, Goupil P. Sequential effects of cadmium on genotoxicity and lipoperoxidation in Vicia faba roots. Ecotoxicology 2011;20:329–36. doi: 10.1007/s10646-010-0582-0 21153701 10.1007/s10646-010-0582-0

[16] Tatagiba SD, Rodrigues FA, Filippi MCC, Silva GB, Silva LC. Physiological responses of rice plants supplied with silicon to Monographella albescens infection. J Phytopathol 2014;162:596–606.

[17] Nascimento KJT, Araujo L, Resende RS, Schurt DA, Silva WLd, Rodrigues FdÁ. Silicon, acibenzolar-S-methyl and potassium phosphite in the control of brown spot in rice. Bragantia 2016;75:212–21.

[18] Nogueira de Moura Guerra AM, Ávila Rodrigues F, Berger PG, Barros AF, Rodrigues da Silva YC, Costa Lima T. Aspectos bioquímicos da resistência do algodoeiro à ramulose potencializada pelo silício. Bragantia 2013;72:292–303.

[19] Van Loon L, Van Strien E. The families of pathogenesis-related proteins, their activities, and comparative analysis of PR-1 type proteins. Physiol Mol Plant Pathol 1999;55:85–97.

[20] van Doorn WG, Ketsa S. Cross reactivity between ascorbate peroxidase and phenol (guaiacol) peroxidase. Postharvest Biol Technol 2014;95:64–9.

[21] Zhang X, Guo M, Guo C, Yang H, Liu C-J. Down-regulation of a kelch domain-containing F-box protein in Arabidopsis enhances the production of (poly) phenols and tolerance to UV-radiation. Plant Physiol 2014:pp. 114.249136. 10.1104/pp.114.249136 PMC4326750 25502410

[22] Song A, Xue G, Cui P, Fan F, Liu H, Yin C, et al The role of silicon in enhancing resistance to bacterial blight of hydroponic- and soil-cultured rice. Scientific Reports 2016;6:24640 doi: 10.1038/srep24640 27091552 10.1038/srep24640 PMC4835757

[23] Wada KC, Mizuuchi K, Koshio A, Kaneko K, Mitsui T, Takeno K. Stress enhances the gene expression and enzyme activity of phenylalanine ammonia-lyase and the endogenous content of salicylic acid to induce flowering in pharbitis. J Plant Physiol 2014;171:895–902. doi: 10.1016/j.jplph.2014.03.008 24913046 10.1016/j.jplph.2014.03.008

[24] Mercado-Blanco J JJ Lugtenberg B. Biotechnological applications of bacterial endophytes. Current Biotechnology 2014;3:60–75.

[25] Halo BA, Khan AL, Waqas M, Al-Harrasi A, Hussain J, Ali L, et al Endophytic bacteria (Sphingomonas sp. LK11) and gibberellin can improve Solanum lycopersicum growth and oxidative stress under salinity. Journal of Plant Interactions 2015;10:117–25.

[26] Wang M, Wu C, Cheng Z, Meng H. Growth and physiological changes in continuously cropped eggplant (Solanum melongena L.) upon relay intercropping with garlic (Allium sativum L.). Frontiers in plant science 2015;6:262 doi: 10.3389/fpls.2015.00262 25964788 10.3389/fpls.2015.00262 PMC4408842

[27] Lamb C, Dixon RA. The oxidative burst in plant disease resistance. Annu Rev Plant Biol 1997;48:251–75. 10.1146/annurev.arplant.48.1.251 15012264

[28] Kombrink E, Schmelzer E. The hypersensitive response and its role in local and systemic disease resistance. Eur J Plant Pathol 2001;107:69–78.

[29] Henkes GJ, Jousset A, Bonkowski M, Thorpe MR, Scheu S, Lanoue A, et al Pseudomonas fluorescens CHA0 maintains carbon delivery to Fusarium graminearum-infected roots and prevents reduction in biomass of barley shoots through systemic interactions. J Exp Bot 2011;62:4337–44. doi: 10.1093/jxb/err149 21561952 10.1093/jxb/err149 PMC3153684

[30] Lavania M, Chauhan PS, Chauhan S, Singh HB, Nautiyal CS. Induction of plant defense enzymes and phenolics by treatment with plant growth–promoting rhizobacteria Serratia marcescens NBRI1213. Curr Microbiol 2006;52:363–8. doi: 10.1007/s00284-005-5578-2 16586018 10.1007/s00284-005-5578-2

[31] Senthilraja G, Anand T, Kennedy J, Raguchander T, Samiyappan R. Plant growth promoting rhizobacteria (PGPR) and entomopathogenic fungus bioformulation enhance the expression of defense enzymes and pathogenesis-related proteins in groundnut plants against leafminer insect and collar rot pathogen. Physiol Mol Plant Pathol 2013;82:10–9.

[32] Bano A, Muqarab R. Plant defence induced by PGPR against Spodoptera litura in tomato (Solanum lycopersicum L.). Plant biology (Stuttgart, Germany) 2017;19:406–12. 10.1111/plb.12535 28004873

[33] Raj TS, Anandeeswari D, Suji H, Joice AA. Role of defence enzymes activity in rice as induced by idm formulations against sheath blight caused by *Rhizoctonia solani* IJAPSA 2016; 02:106–16.

[34] Singh N, Kaur L, Sodhi NS, Sekhon KS. Physicochemical, cooking and textural properties of milled rice from different Indian rice cultivars. Food Chem 2005;89:253–9.

[35] Sharma M. Actinomycetes: source, identification, and their applications. Int J Curr Microbiol App Sci 2014;3:801–32.

[36] Hassan M, Afghan S, Hafeez F. Biological suppression of sugarcane red rot by Bacillus spp. under field conditions. J Plant Pathol 2012;94:325–9.

[37] Hassan MN, Afghan S, Hafeez FY. Suppression of red rot caused by *Colletotrichum falcatum* on sugarcane plants using plant growth-promoting rhizobacteria. BioControl 2010;55:531–42.

[38] Hassan MN, Shah SZ-U-H, Afghan S, Hafeez FY. Suppression of red rot disease by *Bacillus* sp. based biopesticide formulated in non-sterilized sugarcane filter cake. BioControl 2015;60:691–702.

[39] Qin S, Xing K, Jiang J-H, Xu L-H, Li W-J. Biodiversity, bioactive natural products and biotechnological potential of plant-associated endophytic actinobacteria. Appl Microbiol Biotechnol 2011;89:457–73. doi: 10.1007/s00253-010-2923-6 20941490 10.1007/s00253-010-2923-6

[40] Rais A, Shakeel M, Hafeez FY, Hassan MN. Plant growth promoting rhizobacteria suppress blast disease caused by Pyricularia oryzae and increase grain yield of rice. BioControl 2016;61:769–80.

[41] Reeves MW, Pine L, Neilands J, Balows A. Absence of siderophore activity in Legionella species grown in iron-deficient media. J Bacteriol 1983;154:324–9. 6219988 10.1128/jb.154.1.324-329.1983 PMC217462

[42] Sankaranarayanan R, Alagumaruthanayagam A, Sankaran K. A new fluorimetric method for the detection and quantification of siderophores using Calcein Blue, with potential as a bacterial detection tool. Appl Microbiol Biotechnol 2015;99:2339–49. doi: 10.1007/s00253-015-6411-x 25634020 10.1007/s00253-015-6411-x

[43] Yang G, Huang TS. Human face detection in a complex background. Pattern recognition 1994;27:53–63.

[44] Joo H-S, Kumar CG, Park G-C, Kim KT, Paik SR, Chang C-S. Optimization of the production of an extracellular alkaline protease from Bacillus horikoshii. Process Biochem 2002;38:155–9.

[45] Mayerhofer H, Marshall R, White C, Lu M. Characterization of a heat-stable protease of Pseudomonas fluorescens P26. Applied microbiology 1973;25:44–8. 4631436 10.1128/am.25.1.44-48.1973 PMC380733

[46] Wood TM, Bhat KM. Methods for measuring cellulase activities. Methods Enzymol 1988;160:87–112.

[47] Dewi RTK, Mubarik NR, Suhartono MT. Medium optimization of [beta]-glucanase production by Bacillus subtilis SAHA 32.6 used as biological control of oil palm pathogen. Emirates Journal of Food and Agriculture 2016;28:116.

[48] Jabeen Z, Hussain N, Wu D, Han Y, Shamsi I, Wu F, et al Difference in physiological and biochemical responses to salt stress between Tibetan wild and cultivated barleys. Acta physiologiae plantarum 2015;37:180.

[49] Vaikuntapu PR, Dutta S, Samudrala RB, Rao VR, Kalam S, Podile AR. Preferential Promotion of *Lycopersicon esculentum* (Tomato) Growth by Plant Growth Promoting Bacteria Associated with Tomato. Indian J Microbiol 2014;54:403–12. doi: 10.1007/s12088-014-0470-z 25320438 10.1007/s12088-014-0470-z PMC4186933

[50] Anand T, Chandrasekaran A, Kuttalam S, Raguchander T, Prakasam V, Samiyappan R. Association of some plant defense enzyme activities with systemic resistance to early leaf blight and leaf spot induced in tomato plants by azoxystrobin and Pseudomonas fluorescens. Journal of Plant Interactions 2007;2:233–44.

[51] Mayer A, Harel E, Ben-Shaul R. Assay of catechol oxidase—a critical comparison of methods. Phytochemistry 1966;5:783–9.

[52] Sainders JA, McClure JW. Phytochrome controlled phenylalanine ammonia lyase in Hordeum vulgare plastids. Phytochemistry 1975;14:1285–9.

[53] DHINDSA RS, Plumb-Dhindsa P, THORPE TA. Leaf senescence: correlated with increased levels of membrane permeability and lipid peroxidation, and decreased levels of superoxide dismutase and catalase. J Exp Bot 1981;32:93–101.

[54] Hammerschmidt R, Nuckles E, Kuć J. Association of enhanced peroxidase activity with induced systemic resistance of cucumber to Colletotrichum lagenarium. Physiological Plant Pathology 1982;20:73IN977–76IN1082.

[55] Rao KM, Sresty T. Antioxidative parameters in the seedlings of pigeonpea (Cajanus cajan (L.) Millspaugh) in response to Zn and Ni stresses. Plant Sci 2000;157:113–28. 10940475 10.1016/s0168-9452(00)00273-9

[56] Zhang Y-K, Zhu D-F, Zhang Y-P, Chen H-Z, Xiang J, Lin X-Q. Low pH-induced changes of antioxidant enzyme and ATPase activities in the roots of rice (Oryza sativa L.) seedlings. PloS one 2015;10:e0116971 doi: 10.1371/journal.pone.0116971 25719552 10.1371/journal.pone.0116971 PMC4342341

[57] Barillot CD, Sarde C-O, Bert V, Tarnaud E, Cochet N. A standardized method for the sampling of rhizosphere and rhizoplan soil bacteria associated to a herbaceous root system. Annals of microbiology 2013;63:471–6.

[58] Sheridan C, Depuydt P, De Ro M, Petit C, Van Gysegem E, Delaere P, et al Microbial Community Dynamics and Response to Plant Growth-Promoting Microorganisms in the Rhizosphere of Four Common Food Crops Cultivated in Hydroponics. Microb Ecol 2016:1–16. 10.1007/s00248-016-0855-0 27645138

[59] Lethbridge G, Bull A, Burns RG. Assay and properties of 1, 3-β-glucanase in soil. Soil Biol Biochem 1978;10:389–91.

[60] Powell PE, Szaniszlo PJ, Reid CP. Confirmation of occurrence of hydroxamate siderophores in soil by a novel Escherichia coli bioassay. Appl Environ Microbiol 1983;46:1080–3. 16346415 10.1128/aem.46.5.1080-1083.1983 PMC239522

[61] Ashelford KE, Chuzhanova NA, Fry JC, Jones AJ, Weightman AJ. New screening software shows that most recent large 16S rRNA gene clone libraries contain chimeras. Appl Environ Microbiol 2006;72:5734–41. doi: 10.1128/AEM.00556-06 16957188 10.1128/AEM.00556-06 PMC1563593

[62] Zhao Y. Auxin biosynthesis and its role in plant development. Annu Rev Plant Biol 2010;61:49–64. doi: 10.1146/annurev-arplant-042809-112308 20192736 10.1146/annurev-arplant-042809-112308 PMC3070418

[63] Gill SS, Tuteja N. Reactive oxygen species and antioxidant machinery in abiotic stress tolerance in crop plants. Plant Physiol Biochem 2010;48:909–30. doi: 10.1016/j.plaphy.2010.08.016 20870416 10.1016/j.plaphy.2010.08.016

[64] Das K, Roychoudhury A. Reactive oxygen species (ROS) and response of antioxidants as ROS-scavengers during environmental stress in plants. Frontiers in Environmental Science 2014;2:53.

[65] Pusztahelyi T, Holb IJ, Pócsi I. Secondary metabolites in fungus-plant interactions. Frontiers in plant science 2015;6:573 doi: 10.3389/fpls.2015.00573 26300892 10.3389/fpls.2015.00573 PMC4527079

[66] Samalova M, Meyer AJ, Gurr SJ, Fricker MD. Robust anti‐oxidant defences in the rice blast fungus Magnaporthe oryzae confer tolerance to the host oxidative burst. New Phytol 2014;201:556–73. doi: 10.1111/nph.12530 24117971 10.1111/nph.12530

[67] Law JW-F, Ser H-L, Khan TM, Chuah L-H, Pusparajah P, Chan K-G, et al The potential of Streptomyces as biocontrol agents against the rice blast fungus, Magnaporthe oryzae (Pyricularia oryzae). Frontiers in microbiology 2017;8. 28144236 10.3389/fmicb.2017.00003 PMC5239798

[68] Manhas RK, Kaur T. Biocontrol Potential of Streptomyces hydrogenans Strain DH16 toward Alternaria brassicicola to Control Damping Off and Black Leaf Spot of Raphanus sativus. Frontiers in Plant Science 2016;7:1869 doi: 10.3389/fpls.2016.01869 28018402 10.3389/fpls.2016.01869 PMC5159428

[69] Gupta D, Pena L, Romero‐Puertas M, Hernández A, Inouhe M, Sandalio L. NADPH oxidases differentially regulate ROS metabolism and nutrient uptake under cadmium toxicity. Plant, Cell Environ 2016:50 10.1111/pce.12711 26765289

[70] Schmitt F-J, Renger G, Friedrich T, Kreslavski VD, Zharmukhamedov SK, Los DA, et al Reactive oxygen species: re-evaluation of generation, monitoring and role in stress-signaling in phototrophic organisms. Biochimica et Biophysica Acta (BBA)-Bioenergetics 2014;1837:835–48. 24530357 10.1016/j.bbabio.2014.02.005

[71] Ishibashi Y, Yamaguchi H, Yuasa T, Iwaya-Inoue M, Arima S, Zheng S-H. Hydrogen peroxide spraying alleviates drought stress in soybean plants. J Plant Physiol 2011;168:1562–7. doi: 10.1016/j.jplph.2011.02.003 21377755 10.1016/j.jplph.2011.02.003

[72] Sharma P, Jha AB, Dubey RS, Pessarakli M. Reactive oxygen species, oxidative damage, and antioxidative defense mechanism in plants under stressful conditions. Journal of Botany 2012;2012.

[73] van Loon LC, Rep M, Pieterse CM. Significance of inducible defense-related proteins in infected plants. Annu Rev Phytopathol 2006;44:135–62. doi: 10.1146/annurev.phyto.44.070505.143425 16602946 10.1146/annurev.phyto.44.070505.143425

[74] Singh A, Sarma BK, Upadhyay RS, Singh HB. Compatible rhizosphere microbes mediated alleviation of biotic stress in chickpea through enhanced antioxidant and phenylpropanoid activities. Microbiol Res 2013;168:33–40. doi: 10.1016/j.micres.2012.07.001 22857806 10.1016/j.micres.2012.07.001

[75] Sarma BK, Yadav SK, Singh S, Singh HB. Microbial consortium-mediated plant defense against phytopathogens: readdressing for enhancing efficacy. Soil Biol Biochem 2015;87:25–33.

[76] Singh SP, Gaur R. Endophytic Streptomyces spp. underscore induction of defense regulatory genes and confers resistance against Sclerotium rolfsii in chickpea. Biol Control 2017;104:44–56.

[77] Naureen Z, Price AH, Hafeez FY, Roberts MR. Identification of rice blast disease-suppressing bacterial strains from the rhizosphere of rice grown in Pakistan. Crop Protect 2009;28:1052–60.

[78] Foyer CH, Noctor G. Redox homeostasis and antioxidant signaling: a metabolic interface between stress perception and physiological responses. The Plant Cell 2005;17:1866–75. doi: 10.1105/tpc.105.033589 15987996 10.1105/tpc.105.033589 PMC1167537

[79] Chaiharn M, Chunhaleuchanon S, Lumyong S. Screening siderophore producing bacteria as potential biological control agent for fungal rice pathogens in Thailand. World Journal of Microbiology and Biotechnology 2009;25:1919–28.

[80] Sindhu S, Gupta S, Dadarwal K. Antagonistic effect of Pseudomonas spp. on pathogenic fungi and enhancement of growth of green gram (Vigna radiata). Biol Fertility Soils 1999;29:62–8.

[81] Ashwini N, Srividya S. Potentiality of Bacillus subtilis as biocontrol agent for management of anthracnose disease of chilli caused by Colletotrichum gloeosporioides OGC1. 3 Biotech 2014;4:127–36. doi: 10.1007/s13205-013-0134-4 28324440 10.1007/s13205-013-0134-4 PMC3964249

[82] Odier E, Janin G, Monties B. Poplar lignin decomposition by gram-negative aerobic bacteria. Appl Environ Microbiol 1981;41:337–41. 16345706 10.1128/aem.41.2.337-341.1981 PMC243695

[83] Mandal SM, Chakraborty D, Dey S. Phenolic acids act as signaling molecules in plant-microbe symbioses. Plant signaling & behavior 2010;5:359–68. 20400851 10.4161/psb.5.4.10871 PMC2958585

[84] Ali MB, McNear DH. Induced transcriptional profiling of phenylpropanoid pathway genes increased flavonoid and lignin content in Arabidopsis leaves in response to microbial products. BMC Plant Biol 2014;14:84 doi: 10.1186/1471-2229-14-84 24690446 10.1186/1471-2229-14-84 PMC4021374

[85] Shalini P, Sanjay Mohan G, Anil K. Enzymes of phenylpropanoid metabolism involved in strengthening the structural barrier for providing genotype and stage dependent resistance to Karnal bunt in wheat. American Journal of Plant Sciences 2012;2012:261–7.

[86] Zhao Q, Dixon RA. Altering the cell wall and its impact on plant disease: from forage to bioenergy. Annu Rev Phytopathol 2014;52:69–91. doi: 10.1146/annurev-phyto-082712-102237 24821183 10.1146/annurev-phyto-082712-102237

[87] Pellegrini L, Rohfritsch O, Fritig B, Legrand M. Phenylalanine ammonia-lyase in tobacco (molecular cloning and gene expression during the hypersensitive reaction to tobacco mosaic virus and the response to a fungal elicitor). Plant Physiol 1994;106:877–86. 7824656 10.1104/pp.106.3.877 PMC159610

[88] Chandra S, Chakraborty N, Dasgupta A, Sarkar J, Panda K, Acharya K. Chitosan nanoparticles: a positive modulator of innate immune responses in plants. Scientific reports 2015;5:1519. 10.1038/srep15195 PMC4607973 26471771

[89] Mauch-Mani B, Slusarenko AJ. Production of salicylic acid precursors is a major function of phenylalanine ammonia-lyase in the resistance of Arabidopsis to Peronospora parasitica. The Plant Cell 1996;8:203–12. doi: 10.1105/tpc.8.2.203 12239383 10.1105/tpc.8.2.203 PMC161092

[90] Takahashi M-A, Asada K. Superoxide anion permeability of phospholipid membranes and chloroplast thylakoids. Archives of Biochemistry and Biophysics 1983;226:558–66. 6314906 10.1016/0003-9861(83)90325-9

[91] Alscher RG, Erturk N, Heath LS. Role of superoxide dismutases (SODs) in controlling oxidative stress in plants. J Exp Bot 2002;53:1331–41. 11997379

[92] Choi HW, Klessig DF. DAMPs, MAMPs, and NAMPs in plant innate immunity. BMC Plant Biol 2016;16:232 doi: 10.1186/s12870-016-0921-2 27782807 10.1186/s12870-016-0921-2 PMC5080799

[93] Jones JD, Dangl JL. The plant immune system. Nature 2006;444:323 doi: 10.1038/nature05286 17108957 10.1038/nature05286

[94] Bolouri‐Moghaddam MR, Le Roy K, Xiang L, Rolland F, Van den Ende W. Sugar signalling and antioxidant network connections in plant cells. The FEBS journal 2010;277:2022–37. doi: 10.1111/j.1742-4658.2010.07633.x 20412056 10.1111/j.1742-4658.2010.07633.x

[95] Audenaert K, Pattery T, Cornelis P, Höfte M. Induction of systemic resistance to Botrytis cinerea in tomato by Pseudomonas aeruginosa 7NSK2: role of salicylic acid, pyochelin, and pyocyanin. Mol Plant-Microbe Interact 2002;15:1147–56. doi: 10.1094/MPMI.2002.15.11.1147 12423020 10.1094/MPMI.2002.15.11.1147

